# When to Intervene in Acute Necrotizing Pancreatitis: A Narrative Review of the Optimal Timing for Intervention Strategies

**DOI:** 10.3390/medicina60101592

**Published:** 2024-09-27

**Authors:** Daniel Paramythiotis, Eleni Karlafti, Dimitrios Tsavdaris, Alexandros Giakoustidis, Stavros Panidis, Aristeidis Ioannidis, Panos Prassopoulos, Antonios Michalopoulos

**Affiliations:** 1 First Propaedeutic Surgery Department, University General Hospital of Thessaloniki AHEPA, Aristotle University of Thessaloniki, 54636 Thessaloniki, Greece; danosprx@auth.gr (D.P.); tsavdaris@auth.gr (D.T.); st.panidis@gmail.com (S.P.); ariioann@yahoo.gr (A.I.); amichal@auth.gr (A.M.); 2 Emergency Department, University General Hospital of Thessaloniki AHEPA, Aristotle University of Thessaloniki, 54636 Thessaloniki, Greece; 3 First Propaedeutic Department of Internal Medicine, University General Hospital of Thessaloniki AHEPA, Aristotle University of Thessaloniki, 54636 Thessaloniki, Greece; 4 First Surgery Department, University General Hospital of Thessaloniki Papageorgiou, Aristotle University of Thessaloniki, 54636 Thessaloniki, Greece; alexgiakoustidis@gmail.com; 5 Department of Radiology, University General Hospital of Thessaloniki AHEPA, Aristotle University of Thessaloniki, 54636 Thessaloniki, Greece; pprasopo@auth.gr

**Keywords:** acute necrotizing pancreatitis, early intervention, delayed intervention, narrative review

## Abstract

*Introduction*: Acute necrotizing pancreatitis (ANP) is the acute inflammation of pancreatic parenchyma, most commonly due to alcohol abuse or cholelithiasis. The treatment can be either conservative or invasive, including a variety of techniques; however, it has not yet been established if the intervention should be early or if it should be delayed. The aim of this review is to investigate the optimal time for intervention in ANP. *Methods*: A literature search was conducted in PubMed and Scopus from inception until September 2024 for studies reporting the comparison between early and late intervention. *Results*: Early intervention, within 4 weeks of symptom onset, often involves drainage via percutaneous, endoscopic, or combined methods. Delayed intervention occurs after 4 weeks of symptom onset. This can be conducted either surgically or via minimally invasive means. The results of this review reveal that the time of intervention for ANP plays an important role in the prognosis and the course of the disease. In particular, early intervention is associated with higher mortality, which is also the primary clinical outcome. Delayed intervention is also superior regarding secondary clinical outcomes, specifically the complications associated with the intervention. Thus, it is accompanied by fewer episodes of new-onset organ failure, bleeding, gastrointestinal fistula, pancreatic fistula, wound infection, endocrine pancreatic insufficiency, and other complications. Finally, delayed intervention results in shorter stays, both in hospitals and the ICU. *Conclusions*: Delayed intervention is clearly more effective than early intervention and should be preferred. However, early intervention appears to be both safe and effective, and it is feasible.

## 1. Introduction

Acute inflammation of the pancreatic parenchyma is termed acute pancreatitis (AP). It ranges from mild to potentially fatal and is commonly attributed to the enzymatic autodigestion of the organ. It constitutes a prevalent cause of emergency room admissions [1,2]. The clinical symptoms belong to a severe spectrum, primarily characterized by intense and excruciating abdominal pain, which often radiates to the loins and can also be accompanied by nausea or vomiting [3,4].

The etiology involves a wide range of causative factors. The most common of these are cholelithiasis and alcohol. However, AP can also arise from various other causes, including metabolic, infectious, and vascular factors, as well as autoimmune diseases and other underlying conditions. When the cause of AP cannot be identified, then it is called idiopathic [5,6,7]. The underlying etiology of AP serves as the determinant for the ensuing pathophysiological mechanisms [8,9].

Diagnosing AP requires a comprehensive evaluation of clinical presentation and relevant laboratory findings. Measurement of serum amylase within the first 48 h and serum lipase over an extended period holds significant diagnostic value [10,11]. Imaging tests also contribute to the diagnosis of AP; ultrasound can detect gallstones, while multi-detector computed tomography (MDCT) helps in staging the disease. Finally, endoscopic methods can contribute to diagnosis [12,13].

AP can be classified depending on the pathoanatomical picture in terms of whether it is edematous or necrotic. This classification also determines the prognosis, as the edematous form generally has a favorable outcome and often requires conservative management, whereas the necrotic form may necessitate invasive treatment [14,15,16].

Upon hospital admission, the severity of AP is assessed using the Atlanta classification. These criteria take into account the existence of organ failure and major complications to classify it as mild, moderate, and severe [17]. These complications, spanning a diverse array of conditions, can be categorized into either systemic manifestations or localized conditions directly affecting the pancreatic parenchyma. Thus, local ones include the collection of fluid in the peri-alveolar and interstitial tissue, pseudocysts, abscesses and walled-off pancreatic necrosis (WON). Pseudocyst and WON are two different clinical entities, with the first mainly containing fluid along with enzymes, blood, and necrotic tissue, and the second mainly containing necrotic tissue and debris. These differences have an impact on the approach and treatment of these two complications. The systemic ones include kidney failure, circulatory failure, and respiratory distress syndrome in adults [18].

According to the guidelines, after the diagnosis of AP, the prognosis is influenced by various prognostic factors and the patient’s imaging findings [19,20]. Then, treatment follows, with restoration of the patient’s fluids, pain management, nutritional support and prevention of complications, which does not, however, require the administration of prophylactic antibiotic treatment [21,22,23,24].

In cases of necrotizing pancreatitis, the need for intervention is carefully evaluated. This intervention becomes necessary in cases of infected necrotizing pancreatitis, particularly when necrosis has become walled-off, even in the absence of documented infected necrosis with concurrent and persistent organ failure. Intervention may be needed in sterile necrotizing pancreatitis when there are persistent symptoms, biliary obstructions, and disconnected duct syndrome. Numerous treatment options exist for managing acute necrotizing pancreatitis (ANP), and the timing of the intervention also differs. Additionally, interventions may be necessary to drain symptomatic effusions, pseudocysts, or abscesses that may coexist with ANP [25,26,27].

**Figure 1:**
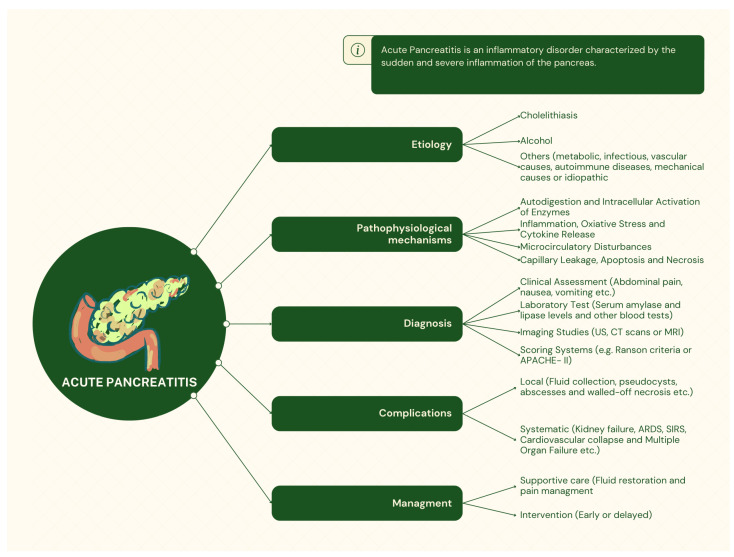
A comprehensive overview of acute pancreatitis. The figure was created using Canva.com.

The various types of interventions for ANP can be categorized into two main groups: surgical and minimally invasive (MI). Surgical interventions for the treatment of ANP include necrosectomy, pancreatic debridement, cystgastrostomy, cystjejunostomy, and other techniques [28,29,30,31]. The MI approach encompasses a range of techniques determined by both the visualization method and the access route. Thus, the laparoscopic technique, the retroperitoneal approach, the endoscopic approach [endoscopic transluminal drainage (ED)], or a combination of these, can be distinguished. The laparoscopic technique presents significantly lower rates of complications, blood loss, and length of stay in the hospital than surgical techniques. However, it presents a longer average operation duration [32]. Necrosectomy can also be performed laparoscopically, with significantly lower morbidity and complications. However, mortality seems to have exceeded 10% [33,34,35]. Finally, the use of invasive radiology, specifically the percutaneous approach for drainage (PCD) of ANP, can also contribute to treatment. This is a method that can be performed shortly after the onset of symptoms using a percutaneous catheter under ultrasound or computerized tomography (CT) guidance [36,37]. A comprehensive overview of AP is depicted in Figure 1. 

The current standard procedure for managing symptomatic pancreatic necrotic fluid collections involves MI step-up interventions. Although traditional guidelines have recommended delaying the drainage of these collections, there has been a recent shift towards earlier, minimally invasive, non-surgical interventions [28,29,30]. This narrative review aims to present and compare the optimal timing of intervention.

## 2. Materials and Methods 

Extensive research was conducted using two databases, PubMed and Scopus, covering publications from inception until September 2024, with a focus solely on articles written in English. The search terms used were “necrotizing pancreatitis AND (early intervention OR delayed intervention)”. Excluded from this review were review articles, letters, comments, and case studies. Studies meeting the inclusion criteria for this review focused on adult patients with ANP, interventions for ANP, and the timing of these interventions. We used the 4-week (28-day) period as a cut-off to distinguish between early and delayed intervention. The aim of this review is to investigate if early intervention in ANP is more effective than late intervention. For this reason, the studies included in this review analyze the clinical outcomes of early and late intervention in patients with AP.

## 3. Results

Using the search terms “necrotizing pancreatitis” and [“early intervention or delayed intervention”], a total of 650 studies were found. Of these, 266 were eliminated for being duplicates, 99 for being reviews, 28 for being case reports, 3 for being protocols, and 12 for being comments and letters. In total, 254 studies remained after being screened. Of these, 236 were eliminated because they failed to meet the inclusion standards of this review. Exclusions were made either because the studies did not specify the timing of intervention or the delayed interventions occurred before the 4-week cut-off or they lacked the primary outcome of interest, which was mortality. Overall, therefore, 18 studies were evaluated for eligibility. To evaluate the ideal timing of intervention in ANP, all were taken into consideration. A total of 18 studies were included. These 18 studies involved 2276 patients with ANP. Of these patients, 1178 were managed with early intervention for ANP and the remaining 1096 with delayed intervention. The early intervention in these studies was below the median of 29 days, reaching a median of 19 days, while the delayed intervention was over a month, reaching a median of 85 days. These days are counted from the onset of symptoms to the intervention. The majority of studies belong to the category of cohort studies, specifically nine of the eighteen studies, while seven studies are case–control studies and two are randomized controlled trials. 

### 3.1. Comparison of Time and Type of Intervention of ANP

The type of intervention varies between studies. The early intervention in these studies was carried out in two different ways. The first is PCD, which was selected by seven studies [38,39,40,41,42,43,44], and the second is ED, which was selected by five studies [45,46,47,48,49]. The remaining six studies [50,51,52,53,54,55] used both the first and second techniques. Delayed intervention was performed either with endoscopically centered step-up interventions [38,40,45,46,47,48,49,51] with surgically centered step-up interventions [39,41,42] with a combination of these [50,52], or finally, with PCD [43,44]. These characteristics are presented in Table 1.

Regarding early interventions in ANP in the studies that used ED, the mortality ranged from 0 to 13.15% [45,46,47,48,49]. On the contrary, in the studies that used PCD [38,39,40,41,42,43,44], the mortality ranged from 2% to 35%, with the majority, i.e., five out of seven, exceeding 10% and only one scoring 0%. This perhaps indicates a potential superiority of ED over PCD in early intervention. Corresponding to delayed intervention, endoscopically centered step-up interventions are associated with slightly lower mortality rates compared to surgically centered step-up interventions.

The definition of early and delayed intervention differs between studies, with the majority [39,41,42,45,46,47,48,49,50,51], however, defining early intervention as an intervention at less than 4 weeks after the onset of the symptoms and the delayed as an intervention at more than 4 weeks after the onset of the symptoms. Nevertheless, Boxhoorn et al., Dost et al., and Veldhuisen [38,40,52] define early as immediate drainage within 24 h. 

### 3.2. Indicators for Intervention and Associated Risk Factors

The indications for intervention include the presence of inflammation, obstruction of either the gastric outlet or the biliary tract, abdominal pain, as well as others, such as weight loss or non-response to conservative treatment. It appears that the most frequent indication is the presence of inflammation, especially in the early intervention group. In the delayed intervention group, pain is also a very common indication. These indications of the included studies are shown in the Supplementary Table S1 [38,39,40,41,42,45,46,47,48,49,50,51,52,53,54,55].

### 3.3. Extent of Pancreatic Necrosis and Underlying Causes

Some of the characteristics of necrosis are also mentioned in these studies. These include the percentage extent as assessed on CT or Magnetic Resonance Imaging (MRI), as well as the diameter of the necrotic collection. In the majority of cases, the extent covers more than 50% both in the early intervention group and in the delayed intervention group. The diameter of the collection varies between studies, and these characteristics are shown in Table 2. The median diameter of the collection seems to be smaller in the early intervention group, which is explained by the progression of the disease until the intervention, and other factors, such as the formation of walled-off necrosis and others.

These studies reported 970 cases in which ANP was due to gallstones. Overall, 394 of these belong to the early intervention group, and 383 belong to the delayed intervention group. In total, 673 incidents are due to alcohol abuse, with most of them being in the delayed intervention group. Finally, 659 cases are attributed to other causes, such as hypertriglyceridemia, hypercalcemia, or idiopathic origins with unknown etiology. The distribution of these etiologies between the two groups appears to be balanced.

### 3.4. Primary and Secondary Outcomes Assessment in the Context of Intervention

The clinical outcomes related to the treatment of ANP can be distinguished into primary and secondary. The primary clinical outcomes are focused on the mortality rates associated with each intervention. Secondary clinical outcomes include complications as well as ICU and hospital stay. Among these complications, the most common are new-onset organic insufficiency, bleeding, perforation, fistula, either gastrointestinal or pancreatic, infection of the injured person, and pancreatic insufficiency, either endocrine or exocrine. In these studies, the primary and secondary outcomes of both early and late intervention were analyzed [38,39,40,41,42,45,46,47,48,49,50,51,52,53,54,55]. Table 3 shows the clinical results of the early intervention group and of the delayed intervention groups.

The data collected and presented suggest a tendency towards the greater success of delayed intervention. This success concerns both primary clinical outcomes and secondary outcomes. Therefore, the findings hint at the possibility that delayed intervention is associated with lower mortality, since 109 deaths occurred out of 1096 cases, while early intervention resulted in 228 deaths out of 1178 cases. Additionally, the possibility of complications in delayed intervention is lower. These are organ failure, bleeding, gastrointestinal fistula, pancreatic fistula, wound infection, and endocrine pancreatic insufficiency. Specifically, 161 cases of organ failure were observed out of a total of 481 in the early intervention as opposed to only 59 out of a total of 307 in the late intervention. Bleeding, which is one of the most common complications, is more prevalent in the early intervention group, with 112 cases out of 666 (16.8%) compared with 96 cases out of 868 (11%) in the late intervention, while gastrointestinal fistula or perforation, another common complication, has 81 cases out of 701 in early intervention compared to 63 cases out of 703 in late intervention. Additionally, pancreatic fistula and endocrine pancreatic insufficiency presented 21 of 341 and 57 of 260 early intervention cases compared with 20 of 375 and 63 of 323 late intervention cases, respectively, also showing prevalence in the early intervention group. Only wound infection complications seem to be more frequent in the delayed intervention group, with 23 cases out of 329 compared to 9 cases out of 206 in early intervention.

## 4. Discussion

In this review, 18 studies that distinguished the outcomes of early versus delayed intervention for ANP were included. In these studies, involving a total of 2276 patients, outcomes were distinguished into primary and secondary. The primary outcome is mortality, which was considered the predominant finding that determines the effectiveness of the two types of interventions (early vs. delayed). Mortality ranges from 0% to 34.96% in the early intervention group and from 0% to 32.25% in the delayed intervention group. Secondary outcomes include both complications and hospital and intensive care unit stays, as well as the need for reoperation. Therefore, ANP necessitates comprehensive management, encompassing various aspects such as treatment modalities and the timing of intervention. Generally, delayed intervention yields superior primary and secondary outcomes, making it the preferred approach for managing ANP. 

**Figure 2:**
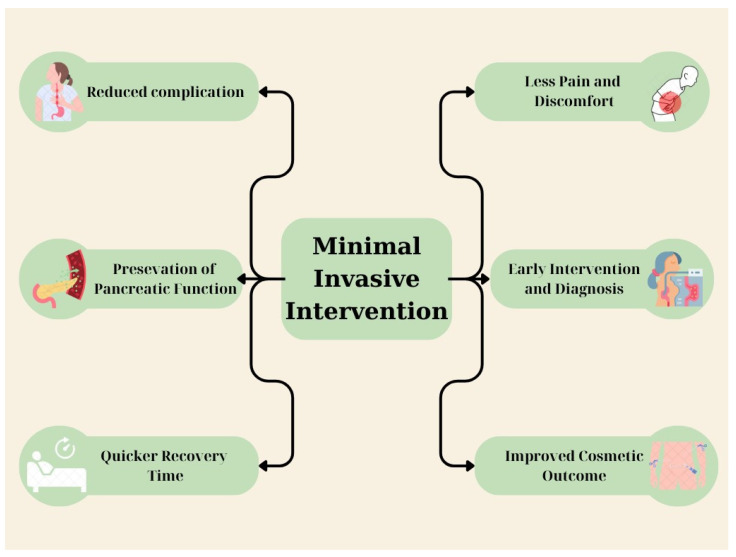
This figure demonstrates the superior benefits of minimally invasive approaches over traditional surgery in the management of acute pancreatitis. The figure was created using Canva.com.

As for intervention types, there are various techniques for the treatment of ANP. All of these can be classified into two major categories: MI and open surgical methods (Figure 2). Two large clinical studies showed the highest effectiveness of MI methods, i.e., endoscopic necrosectomy, PCD, and endoscopic “step-up” therapy [31,56]. In addition, these results are confirmed by other clinical studies, such as van Brunschot et al., which showed that MI surgical necrosectomy and endoscopic necrosectomy are accompanied by lower mortality (OR, 0.53; 95% CI 0.34 to 0.84; *p* = 0.006) and (OR, 0.20; 95% CI 0.06 to 0.63; *p* = 0.006), respectively. It was also found that the reduced mortality of MI methods also applies to high-risk patients with 3/40 vs. 12/40 [57]. Luckhurst et al. also compared mortality between the two types of procedures over a period of one year, as well as the occurrence of complications. The findings indicate higher mortality after 12 months in the surgical approach (15% vs. 3%) as well as higher rates of organ failure. However, bleeding episodes were more common in the MI management of ANP [58]. Additionally, it was found that the ED approach is superior to laparoscopic or video-assisted retroperitoneal debridement as it is accompanied by a lower risk of fistula formation and other complications. The data also suggest having a lower cost as an operation [59]. On the contrary, no difference in cost has been found regarding MI and open surgical methods in general [60]. Drainage may not be possible in a very small percentage of patients, either because of lack of access or because of the contents of the collection [61].

Another surgical technique that seems to contribute a lot to limiting complications, especially mortality, is the step-up technique. It is a technique that begins with mainly PCD and is accompanied by MI retroperitoneal necrosectomy [62]. In this technique, PCD is employed as a method to temporize the situation to allow for delayed intervention with the goal of optimizing outcomes. The four-stage step-up technique has also been proposed, which additionally includes PCD for residual infections and conventional open pancreatic necrosectomy [63]. By using the step-up technique, both the best primary clinical results and the secondary ones are achieved. At the same time, the length of stay in the hospital is significantly reduced. Finally, the step-up technique requires a lower average intervention time from the onset of symptoms. These results were also confirmed by long-term follow-up, which showed a lower need for reoperation [58,62,63,64,65]. It was also found that among the step-up techniques, endoscopic means seem to prevail over surgery, as it is accompanied by lower rates of fistula formation and lower rates of need for reoperations [66].

In the context of the PCD of necrotic collections, this technique is typically applied more frequently within four weeks of the onset of symptoms. It is primarily utilized for inflammation or symptomatic fluid collections. Consequently, PCD is not usually chosen for acute peripancreatic fluid collections due to their tendency to undergo spontaneous resolution, unlike acute necrotic collection (ANC). Therefore, the use of PCD catheters significantly contributes to the avoidance of surgery [67,68]. At the same time, it can also lead to the complete treatment of ANP [43,68,69]. However, the success rate of PCD seems to vary. These discrepancies can be due either to the type of collection (pancreatic pseudocyst has higher success rates) or to management and technique. An important parameter for PCD is the degree of liquification. More solid materials/debris in a collection, implicating the PCD, influence both the complexity of the procedure and the outcome. MRI and US are the modalities of choice in assessing the number of solid materials/debris in a collection, while CT underestimates their presence. Frequent monitoring and regular adjustments, including increasing the catheter diameter, when necessary, appear to enhance the success of the intervention. A larger catheter may be necessary in the case of ANC or WON as the components of the necrotic effusions are thicker and more solid. At the same time, the lower success rates in ANC, apart from the size of the components, are also due to factors, such as associated multiorgan failure and shock, and the presence of central pancreatic necrosis that disrupts the pancreatic duct with the continuous leakage of pancreatic enzymes [43].

Gupta et al. [55] demonstrated that regardless of the baseline severity of ANP, the timing of drainage or the presence of organ failure, percutaneous placement of the large-sized catheter at the outset may result in reduced hospital stay times as well as readmissions. Catheters were distinguished into smaller than 12 F and larger than 12 F. In fully liquefied collections or with minimal debris, 10–12 F catheters might be adequate for drainage. However, in collections with small-to-moderate solid material/debris, 12–16 F catheters are required, while in the presence of abundant solid materials, catheters larger than 16 F might be necessary. Additionally, Šileikis et al. [70] suggest that any surgical attempt to treat ANP should commence with MI techniques. Simultaneously, they emphasize that in case of failure of more than one organ, the operation should not be delayed more than 4 weeks.

Regarding the ED of ANP, Chantarojanasiri et al. suggested that this can be carried out safely within the first 4 weeks as long as it is encapsulated [49,71]. In immature encapsulation, perforation is one of the biggest and most frequent complications [49]. Also, a combination of ED and PCD is another safe option, even when it is performed early [72]. The challenge of this technique is that the use of a percutaneous catheter before ED can make the latter difficult due to the presence of air and the existence of only solid necrotic material [72]. Finally, the use of lumen-apposing metal stents seems to be able to increase the success of ED [73].

ED in ANP can be performed using plastic double pigtail stents, fully covered self-expanding metal stents, preferably using lumen-apposing fully covered self-expanding metal stents (LAMSs). The latter seems to have many advantages over the others. Some of them concern the larger diameter, which leads to more efficient drainage and shorter hospital stays [74]. The use of LAMSs was more frequently indicated in cases of WON and pseudocyst formation [75]. The technical success of the operation exceeds 97%, while the clinical success exceeds 80% [75,76,77,78]. Another advantage of this technique is that the intervention does not require a long period of time. As for adverse events, sepsis and the migration of the stent appear more often in the early stages, while occlusion, infection and esophageal fistula appear as delayed events [75,78]. However, the use of LAMSs is a particularly expensive method compared to the rest available [77].

**Figure 3:**
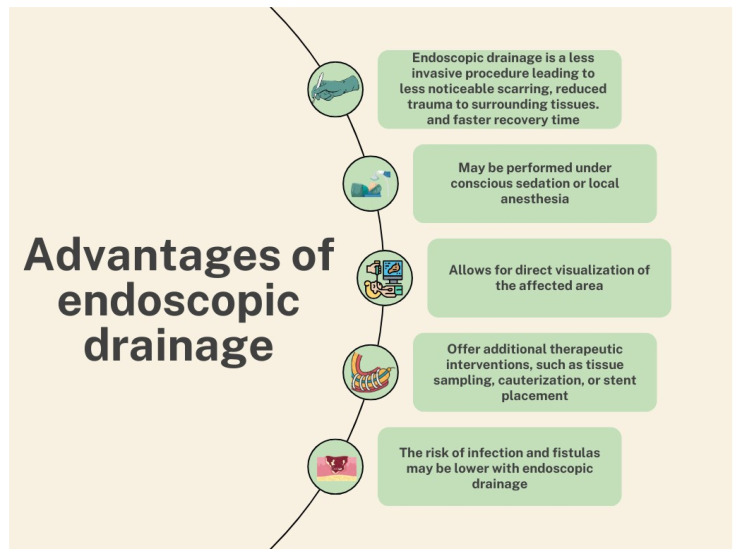
Advantages of endoscopic drainage. The figure was created using Canva.com.

Hence, regarding ED and PCD (Figure 3), there are both advantages and disadvantages. Initially, in ED, there is less risk of infection due to the different routes used, as well as fistula formation. It also does not require moving the patient and does not require general anesthesia [48,50,62]. However, ED depends on the location of the necrotic collection and cannot be carried out independently of it [48,50,62]. Another factor that hampers the success of ED is the presence of solid components in necrotic collections, as it is associated with an increased risk of complications, especially when they exceed 40% [79].

Another difference in treatment options lies in the quality of life that each treatment provides. Conservative treatment and MI methods provide the best quality of life after treatment [59,65,80]. The quality of life after treatment is related to the clinical results of each option. The clinical outcomes that greatly affect the quality of life are endocrine or exocrine pancreatic insufficiency, postoperative hernia as well as the recurrence of pancreatitis [59,65,80]. All primary and secondary clinical outcomes affect quality of life. Poor quality of life is associated with alcoholic pancreatitis [81]. 

Podda et al. [82] proposed an algorithm using artificial intelligence that can direct the appropriate management of acute biliary pancreatitis. Specifically, out of 10 research questions, seven elements of the bundle were identified. According to them, in acute biliary pancreatitis, antibiotic treatment is discouraged, and laparoscopic cholecystectomy within 14 days and ERCP within 2–3 days are recommended. In mild AP, a full solid diet is recommended. In cases when ANP requires surgical intervention, the endoscopic step-up method should be the first course of action [82]. Li et al. [83] presented a nomogram to predict the likelihood of an MI step-up intervention’s success. It can forecast if the intervention will be successful or not based on seven separate parameters. An extrapancreatic necrosis collection found in the small bowel mesentery, APACHE II score, early spontaneous bleeding, platelet and granulocyte decline during the first four weeks, fungal infection, and computed tomography severity index (CTSI) are these factors [83].

Due to the poorer primary and secondary outcomes associated with early intervention, it should only be selected when there are appropriate indications. These concern the occurrence of serious complications during the first four weeks due to systemic inflammation [84]. This systemic inflammation, driven by the release of numerous enzymes and inflammatory cytokines, can exacerbate the patient’s condition, leading to new-onset organ failure or the worsening of pre-existing organ failure, among other complications [85]. Additionally, in some patients, complete encapsulation of the necrotic pancreatic collection may occur within the first three weeks, rendering any delay in treatment unnecessary [86,87]. Moreover, in these patient categories, early intervention does not appear to result in higher complication rates or greater mortality compared to delayed intervention [41].

Indications for early intervention in ANP include hospital admission with elevated levels of four parameters: neutrophils, CRP, PCT, and IL-6 [39]. In addition, the value of Hgb was found to be lower in these patients [39]. Finally, no significant differences were observed in the levels of WBC, Hct, and Alb [39].

**Figure 4:**
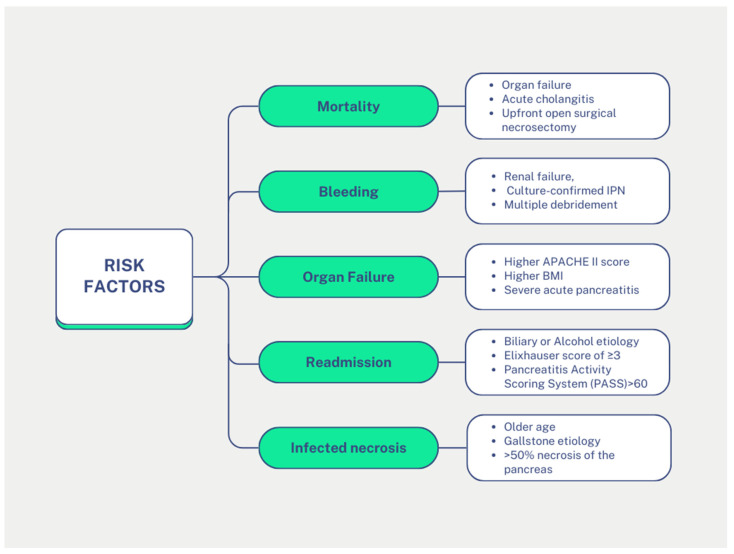
Comprehensive overview of acute pancreatitis risk factors. The figure was created using Canva.com.

There are many prognostic factors concerning the development of ANP or its appearance in the first stage (Figure 4). More specifically, in cases of ANP, factors such as advanced age, the presence of shock, and a high APACHE II score appear to be significant predictors of mortality. Additionally, a prolonged hospital stay is also associated with an increased risk of mortality [88]. A high APACHE II score appears to significantly influence the likelihood of organ failure, alongside the patient’s body weight and the severity of ANP [88]. The severity of ANP seems to be caused by a variety of factors; these initially include the laboratory findings, which include high levels of haematocrit, C-reactive protein, blood urea nitrogen/creatinine, cytokines, and others. Also, the depicted findings can contribute as prognostic factors, specifically a CT scan, which discloses and quantifies the degree of pancreatic parenchyma necrosis. Finally, there are many classification systems for AP, such as the APACHE II and the Ranson score [89]. In patients who underwent endoscopic necrosectomy, the occurrence of bleeding seems to be predicted by factors such as renal failure, culture-confirmed infectious pancreatic necrosis, and multiple debridement procedures [90].

**Figure 5:**
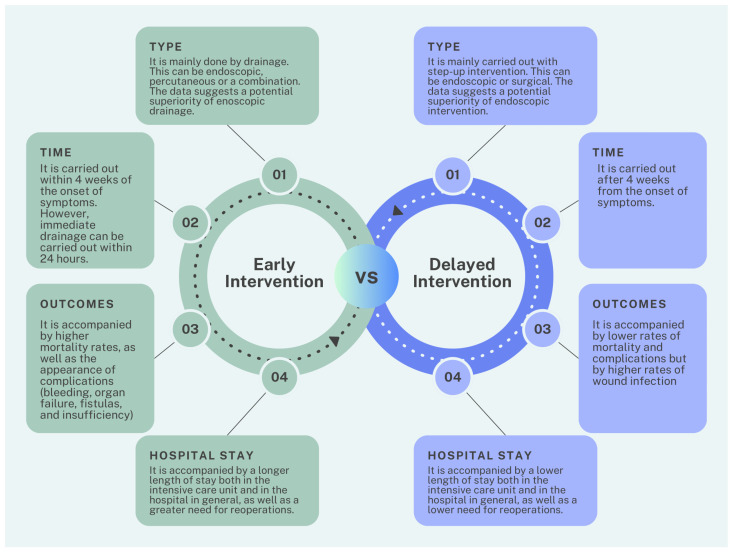
Comparative of early vs. delayed intervention in acute pancreatitis. The figure was created using Canva.com.

Differences between early and delayed treatment are observed in several areas (Figure 5), but particular focus and scrutiny are warranted for the difference between clinical outcomes and hospital stay. In this review, 18 studies [38,39,40,41,42,43,44,45,46,47,48,49,50,51,52,53,54,55], encompassing a total of 2276 patients, were included to compare the outcomes of early versus delayed intervention for ANP. The outcomes in these studies were categorized into primary and secondary. The primary outcome was mortality, which was considered the key determinant of the effectiveness of the two types of interventions (early vs. delayed). Mortality ranges from 0% [45,53] to 34.96% [50] in the early intervention group and from 0% [43,47] to 40% [42] in the delayed intervention group. Secondary outcomes include both complications and hospital and intensive care unit stays, as well as the need for reoperation.

The increased failure of early compared to delayed intervention is due to the poorly formed capsule wall as well as the increased proportion of solid necrotic debris compared to a well-formed WON in the late phase that has mainly fluid content. This leads to the need for many reoperations [47]. According to the revised Atlanta criteria, it takes more than 4 weeks to encapsulate the necrotic collection of the pancreas. Additionally, the liquefaction and encapsulation of the necrotic collection contribute to a clearer demarcation between necrotic and viable tissue. These characteristics make late intervention easier to perform compared to early intervention, which necessitates a highly experienced interventional team to ensure successful outcomes [26,91,92]. However, according to Bomman et al., the exact time period of complete encapsulation may vary from patient to patient. Therefore, CT or MRI scans are valuable tools for precisely determining the extent of encapsulation and the location of the necrotic collection. EUS can also help determine the position of the necrotic effusion relative to the stomach. Finally, a percutaneous shunt sonogram can also help assess complete encapsulation [51,93]. The time until the intervention is covered by conservative treatment, which may include fluid replacement, nutritional support, prevention of complications, and stays in intensive care units [26,94,95].

Another important advantage of the late intervention for ANP, besides low mortality and fewer complications, is the possibility of treating AP conservatively. Conservative treatment is afforded the necessary time to take effect. These findings were confirmed by the POINTER study, in which 39% of patients assigned to the late intervention group were treated with antibiotics alone, with 35% of patients surviving the trials’ initial 6-month follow-up [40,52]. These findings underscore the potential of antibiotic therapy in managing ANP. However, advancing antibiotic development is crucial to optimize their efficacy. Additionally, there is a need to formulate antibiotic regimens tailored to the specific clinical context and patient needs. Specifically, Timmerhuis et al. showed that 48% of patients received the wrong treatment, as it was based only on empiric broad-spectrum antibiotic treatment based on the identified microorganisms. Also, special attention must be paid to avoid the misuse of antibiotics, which can lead to resistance. To mitigate this risk, antibiotic administration should be based on culture results adjusted by imaging-guided fine needle aspiration (FNA) [96,97].

Mortality appears to be significantly affected by organ failure. Mortele et al. showed that the presence of multiple organ failure is the most important outcome indicator [98]. Also, organ failure appears to correlate with the presence of infection and the extent of the necrotic area [99,100]. So, it seems that the percentage of deaths with ANP due to organ failure reaches 50%. Among these, early persistent organ failure carries the worst prognosis compared to transient organ failure. This difference in outcomes is attributed to the distinct pathophysiological mechanisms underlying the two types of organ failure. Specifically, in the early stage, systemic inflammation and the widespread release of cytokines form the pathophysiological basis of organ failure, whereas, in the later stage, organ failure typically results from sepsis [101,102,103,104]. Another factor influencing the outcome of the intervention is the presence of pancreatic parenchymal necrosis, which is associated with higher mortality. In contrast, the presence of only peripancreatic necrosis is linked to lower mortality. However, despite the lower mortality in the second category, complications were more frequent, and there was a greater need for reoperation [105].

Another factor associated with increased mortality is walled-off necrosis infection. Specifically, infected WON compared to sterile presents greater mortality and complications. Fistula formation belongs to these complications Additionally, an extended stay in the ICU and a prolonged hospital admission are indicative of WON. Finally, there was a greater need for drains in these patients [106]. Also, for sterile WON, MI surgical and endoscopic cyst gastrostomy have been proposed for its treatment with lower cost and LOS related to surgical treatment [107].

Concerning the occurrence of bleeding as a complication of MIS, it appears to be strongly linked to a significant rise in mortality rates. Consequently, it necessitates meticulous attention and specialized care. Intravascular embolization stands out as a viable treatment option for addressing this issue [108].

One of the complications of ANP is post-pancreatitis diabetes mellitus or type 3c DM. It is a chronic disease that significantly affects the patient’s quality of life [109,110,111]. Within the first three years following severe ANP, type 3c DM appears to develop in around one out of every four individuals [112,113]. According to Yu et al. [110], there are several characteristics that increase the risk of acquiring diabetes, including age, gender, etiology, APACHE II score, the severity of ANP, organ failure, pancreatic necrosis, and history of smoking and drinking. The management of this type of diabetes includes various methods. First, because pancreatitis severity and prevention are closely correlated, it is important to focus on preventing the development of severe pancreatitis, but the patient’s lifestyle should also be taken into consideration. Considering the patients’ poorer glycemic control, the remaining course of treatment is similar to that for type 2 diabetes [111,114].

Finally, it is important to acknowledge that the timing of the intervention may be beyond the clinician’s control, as it is often dictated by the natural progression of the disease in individual patients.

Further investigation is imperative to comprehensively elucidate the management intricacies of ANP. Initially, a full, thorough investigation of the methods of treating AP is required in all areas (time, techniques, etc.). The potential efficacy of LAMSs in treating AP appears promising; nevertheless, comprehensive research is necessary to establish a reliable protocol for their routine use and to mitigate associated costs. Research should be conducted to enhance interventions in the early stages of encapsulation for more effective and timely treatments. The contribution of early cholecystectomy to biliary pancreatitis also requires investigation. Further study is also important for the improvement of the conservative treatment to reach the point of replacing the intervention. It is recommended that more investigation be carried out in this area, particularly to determine the precise function of antibiotics in the treatment of infected necrosis. The potential role of MRI in the decision-making process should be further elucidated. It is important to have solid evidence that MRI is superior to CT in accurately assessing the degree of pancreatic necrosis, distinguishing between pancreatic and peripancreatic necrosis, assessing the degree of liquefaction, or evaluating the process of wall formation in WON, etc. This information would enhance our understanding of the optimal time of intervention during ANP.

Complications also require further investigation. Investigating and elucidating the pathophysiology of type 3 diabetes following AP is also an imperative step in understanding it and designing more effective prevention and treatment. To direct research into these putative pathogenic processes, dynamic assessments of insulin production, resistance, and pancreatic and incretin hormone response are required. Treatment strategies for AP-related type-3 diabetes can be emphasized with the aid of dynamic assessments of glucose homeostasis. Simultaneously, it is essential to explore the timing of pancreatic endocrine function recovery following ANP and determine methods for assessing it, such as through the examination of HbA1c and C-peptide levels. The long-term follow-up data in studies comparing the effectiveness of early versus late interventions are also limited.

## 5. Conclusions

In conclusion, treatment options encompass a spectrum of techniques, spanning both conservative and invasive approaches, with the flexibility to be applied either early or through delayed interventions. Delayed intervention appears to be more effective than early intervention, as it is associated with lower mortality rates and fewer complications. Additionally, delayed intervention is linked to shorter hospital and intensive care unit stays, as well as a reduced need for reoperations. Therefore, it should be preferred to early intervention. Early intervention should be chosen only when there are appropriate indications to support this choice, which include the occurrence of complications such as organ failure as well as complete capsule wall formation in less than 3 weeks. In all other cases, late intervention is preferable.

## Figures and Tables

**Table 1:** Study Characteristics of included studies.

Study ID	Location	Study Type	Total Subjects	No of Patients Early/Delayed	Early Intervention (Time from Symptom Onset to Intervention)	Delay Intervention (Time from Symptom Onset to Intervention)	Type of Early Intervention	Type of Delayed Intervention
Jagielski et al., 2022 [48]	Poland	Cohort study	71	25/46	Median 16.4 days	Median 74.5 days	ED	Endoscopically centered step-up interventions
Rana et al., 2021 [47]	India	Case–control study	170	34/136	Mean 24 days	Mean 75 days	ED	Endoscopically centered step-up interventions
Boxhoorn et al., 2021 [52]	The Netherlands	Randomized controlled trial	104	55/49	Median 24 days	Median 34 days	PCD OR ED	Endoscopically centered step-up interventions
Trikudanathan et al., 2018 [46]	USA	Case–control study	193	76/117	Median 20 days	Median 78 days	ED	Endoscopically centered step-up interventions
Chantarojanasiri et al., 2018 [49]	Japan	Cohort study	35	12/23	Μedian 23 days	Μedian 85 days	ED	Endoscopically centered step-up interventions
Dost et al., 2022 [38]	Pakistan	Cohort study	130	65/65	ΝΜ	ΝΜ	PCD	Endoscopically centered step-up interventions
Ke et al., 2021 [42]	China	Randomized controlled trial	30	15/15	Median 15.5 days	Median 22 days	PCD	Surgically centered step-up interventions
Lu et al., 2022 [39]	China	Case–control study	98	43/55	Mean 15.26 days	Mean 50.86 days	PCD	Surgically centered step-up interventions
Zhang et al., 2022 [41]	China	Case–control study	131	100/31	Median 19 days	Median 33 days	PCD	Surgically centered step-up interventions
Oblizajek et al., 2020 [45]	USA	Case–control study	38	19/19	Median 23 days	Median 64 days	ED	Endoscopically centered step-up interventions
Santvoort et al., 2011 [50]	The Netherlands	Cohort study	142	143/99	<29 days	>29 days	PCD OR ED	Surgically centered step-up interventions AND endoscopically centered step-up interventions
Bomman et al., 2023 [51]	USA	Case–control study	212	39/173	Median 22 days	Median 52 days	PCD AND ED	Endoscopically centered step-up interventions
Veldhuisen et al., 2023 [40]	The Netherlands	Randomized controlled trial	88	47/41	NM	NM	PCD	Endoscopically centered step-up interventions
Gupta et al., 2021 [55]	India	Cohort study	146	90/54	NM	NM	PCD OR ED	Endoscopically centered step-up interventions
Guo et al., 2014 [54]	China	Cohort study	223	136/87	Median 20 days	Median 49 days	PCD OR Open OR Retroperitoneal pancreatic necrosectomy	PCD OR Open OR Retroperitoneal pancreatic necrosectomy
Woo et al., 2017 [53]	Australia	Cohort study	30	7/23	NM	NM	NM	NM
Mallick et al., 2018 [44]	India	Cohort study	375	258/117	NM	NM	PCD	PCD
Ganaie et al., 2021 [43]	India	Cohort study	60	24/16	NM	NM	PCD	PCD

**Table 2:** Characteristics of pancreatic necrosis and etiology.

Study ID	Early Intervention				Delayed Intervention				Etiology		
	Extent of Pancreatic Necrosis			Median Diameter (Range), cm	Extent of Pancreatic Necrosis			Median Diameter (Range), mm	Gallstones (Early/Delayed)	Alcohol Abuse (Early/Delayed)	Other or Idiopathic (Early/Delayed)
	<30%	30–50%	>50%		<30%	30–50%	>50%				
Jagielski et al., 2022 [48]	0 (0%)	0 (0%)	24 (100%)	17.8 (8.8–32)	0 (0%)	9 (19.6%)	37 (80.4%)	11.7 (6.8–24.7)	NM	47	24
Rana et al., 2021 [47]	NM			12.3	NM			10.5	36 (8/28)	116 (22/94)	69 (27/42)
Boxhoorn et al., 2021 [52]	35 (64%)	8 (15%)	12 (22%)	NM	33 (67%)	7 (14%)	9 (18%)	NM	65 (34/53)	15 (8/7)	NM
Trikudanathan et al., 2018 [46]	NM			17.5 (13.4– 23.4)	NM			14 (9.2–18.6)	87 (34/53)	49 (19/30)	47 (23/24)
Chantarojanasiri et al., 2018 [49]	NM			9.4 (4–18)	NM			12.3 (1.7–25)	15 (8/7)	6 (2/4)	9 (1/8)
Dost et al., 2022 [38]	NM								81 (42/39)	20 (10/10)	29 (13/16)
Ke et al., 2021 [42]	3 (20%)	6 (40.0%)	6 (40.0%)	5.78 (5.11 to 7.88)	2 (13.3%)	3 (20%)	10 (66.7%)	6.11 (4.24 to 9.44)	13 (7/5)	NM	18 (8/18)
Lu et al., 2022 [39]	12 (27.91)	16 (37.21)	15 (34.88)	NM	20 (36.36)	20 (36.36)	15 (27.27)	NM	50 (21/19)	2 (0/2)	46 (22/24)
Zhang et al., 2022 [41]	NM								66 (51/15)	NM	65 (49/16)
Oblizajek et al., 2020 [45]	NM			16 (7–24)	NM			15 (5–22)	18 (8/10)	1 (0/1)	19 (11/8)
Santvoort et al., 2011 [50]	NM								112	55	75
Bomman et al., 2023 [51]	NM			13.8 ± 3.7	NM			12.8 ± 4.5	107 (24/83)	50 (4/45)	60 (10/50)
Gupta et al., 2021 [55]	NM								48	72	24
Guo et al., 2014 [54]	52 (38%)	37 (27%)	45 (35%)	NM	37 (42%)	22 (25%)	29 (33%)	NM	108 (67/41)	24 (13/11)	91 (56/35)
Woo et al., 2017 [53]	NM								3	13	14
Mallick et al., 2018 [44]	NM								133 (88/45)	193 (134/59)	49
Ganaie et al., 2021 [43]	NM								30	10	20

**Table 3:** Primary and secondary clinical outcomes for early and delayed intervention group.

	Primary Outcome		Secondary Outcomes															
Study ID	Mortality [*n* (%)]		Persistent Organ Failure		Bleeding		Gastrointestinal Fistula		Pancreatic Fistula		Wound Infection		Endocrine Pancreatic Insufficiency		ICU Stay		Total Hospital Stays	
	Early	Delayed	Early	Delayed	Early	Delayed	Early	Delayed	Early	Delayed	Early	Delayed	Early	Delayed	Early	Delayed	Early	Delayed
Jagielski et al., 2022 [48]	4%	4.34%	-	-	16%	13%	0%	2.17%	-	-	-	-	28%	30.4%	-	-	-	-
Rana et al., 2021 [47]	5.88%	0%	-	-	20.59%	1.47%	-	-	-	-	-	-	-	-	-	-	-	-
Boxhoorn et al., 2021 [52]	12.72%	10.2%	25.45%	22.45%	14.54%	20.40%	9%	8.16%	10.9%	8.16%	0%	2%	2%	20.4%	12	12	59	51
Trikudanathan et al., 2018 [46]	13.15%	4.27%	-	-	10.53%	9.45%	32.89%	20.5%	-	-	-	-	19.73%	21.36%	2.5	0	41.93	33.83
Chantarojanasiri et al., 2018 [49]	8.33%	4.34%	-	-	25%	0%	0%	17.39%	-	-	-	-	-	-	-	-	27.5	31
Dost et al., 2022 [38]	15.38%	10.76%	-	-	15.38%	20%	10.76%	10.76%	12.7%	10.77%	10.76%	15.38%	-	-	-	-	12.9	16.7
Ke et al., 2021 [42]	2%	40%	-	-	13.13%	13.33%	0%	13.33%	6.67%	6.67%	-	-	-	-	-	-	35.36	30.91
Lu et al., 2022 [39]	13.95%	10.9%	-	-	4.65%	7.27%	4.65%	3.63%	-	-	-	-	25.6%	12.72%	-	-	40.28	47.76
Zhang et al., 2022 [41]	35%	32.25%	49%	38.7%	35%	35.49%	29%	12.9%	2%	0%	-	-	-	-	30	22	45.25	45.34
Oblizajek et al., 2020 [45]	0%	5.26%	-	-	5.26%	15.79%	-	-	-	-	-	-	10.5%	5.26%	1	0	25.69	10.27
Santvoort et al., 2011 [50]	34.96%	15.15%	51.74%	27.27%	-	-	-	-	-	-	-	-	-	-	-	-	-	-
Bomman et al., 2023 [51]	5.12%	5.17%	-	-	10.26%	14.37%	0%	1.15%	6.78%	4.6%	25.64%	5.74%	-	-	-	-	-	-
Veldhuisen et al., 2023 [40]	14.89%	12.19%	8.51%	4.87%	2.13%	0%	2.12%	24.39%	2.1%	0%	2.12%	4.87%	26.2%	16.2%	-	-	-	-
Gupta et al., 2021 [55]	17.78%	14.81%	-	-	-	-	-	-	-	-	-	-	-	-	-	-	-	-
Guo et al., 2014 [54]	21%	10%	15%	8%	20%	9%	9%	14%	-	-	-	-	-	-	-	-	-	-
Woo et al., 2017 [53]	0	17.39%	-	-	-	-	-	-	-	-	-	-	-	-	-	-	137	66
Mallick et al., 2018 [44]	18.99%	13.67%	-	-	-	-	-	-	-	-	-	-	-	-	-	-	22.0 ± 13.6	22.9 ± 12.6
Ganaie et al., 2021 [43]	4.17%	0	-	-	-	-	-	-	-	-	-	-	-	-	-	-	-	-

## Supplementary Materials

The following supporting information can be downloaded at https://www.mdpi.com/article/10.3390/medicina60101592/s1, Table S1: Indicators for Intervention and Associated Risk Factors.
