# Supplementary material for: When to Intervene in Acute Necrotizing Pancreatitis: A Narrative Review of the Optimal Timing for Intervention Strategies
Source: Medicina (Kaunas). 2024 Sep 27;60(10):1592. doi: 10.3390/medicina60101592 (PMC11509130; doi:10.3390/medicina60101592)
Supplement: Supplementary file 1 [file medicina-60-01592-s001.zip › medicina-3218373-supplementary.pdf]

Supplementary Table S1. Indicators for Intervention and Associated Risk Factors

| Study ID | Infection | | Obstruction [Gastric outlet/Biliary tract] | | Pain | | Other indications | |
|---|---|---|---|---|---|---|---|---|
| | Early intervention | Delayed intervention | Early intervention | Delayed intervention | Early intervention | Delayed intervention | Early intervention | Delayed intervention |
| **Jagielski et al., 2022 [51]** | 16 (64.0%) | 20 (43.5%) | NM | NM | 0 (0.0%) | 15 (32.6%) | 14 (56%) | 32(76.1%) |
| **Trikudanathan et al., 2018 [49]** | 69 (90.8%) | 46 (39.3%) | 6 [4 (5.3%)/ 2 (2.6%)] | 15 [(12.8%)/ 5 (4.3%)] | 0 | 45 (38.5%) | 1 (1.3) | 6 (5.1%) |
| **Chantarojanasiri et al., 2018 [52]** | 8(66.7%) | 16(69.5%) | NM | NM | 3(25%) | 3(13%) | 1 (8.3%) | 4(17.4%) |
| **Lu et al., 2022 [42]** | 37 (86.05) | 31 (56.36) | 3 (6.98) | 16 (29.09) | 0 (0) | 4 (7.27) | 3 (6.98) | 4 (7.27) |
| **Zhang et al., 2022[44]** | 90 (90.0%) | 21 (67.7%) | 2 (2.0%) | 6 (19.4%) | NM | NM | 8 (8.0%) | 4 (12.9%) |
| **Oblizajek et al., 2020 [48]** | 13 (68) | 13 (68) | 0(0) | 1 (5) | 9 (47) | 12 (63) | 15(74%) | 10(53%) |
| **Bomman et al., 2023[54]** | 27 (69.2) | 82 (47.1) | 9 (23.1) | 34 (19.5) | 6 (15.4) | 70 (40.2) | NM | NM |
| **Guo et al., 2014[57]** | 77(57) | 57(66) | NM | NM | NM | NM | NM | NM |
